# Qualitative analysis of elite football players’ causal attribution to not playing: A perspective of attribution theory

**DOI:** 10.1371/journal.pone.0325174

**Published:** 2025-06-05

**Authors:** Mehmet Ulas, İlhan Adilogullari, Ender Şenel

**Affiliations:** 1 Physical Education and Sport, Faculty of Sport Sciences, Burdur Mehmet Akif Ersoy University, Burdur, Türkiye; 2 Sport Management, Faculty of Sport Sciences, Canakkale Onsekiz Mart University, Çanakkale, Türkiye; 3 Physical Education and Sport, Faculty of Sport Sciences, Mugla Sitki Kocman University, Kötekli, Türkiye; Paris School of Business, FRANCE

## Abstract

This study explores the causal attributions of professional football players who experience reduced playing time or exclusion from the squad, focusing on the psychological and relational consequences of these experiences. Using a qualitative thematic analysis approach, semi-structured interviews were conducted with 16 elite football players from Turkey’s top league, all of whom had represented the national team. The findings reveal that players predominantly attribute their exclusion to coach preferences and subjective decisions, rather than their performance. This attribution process often leads to negative emotional outcomes, including feelings of exclusion, loss of motivation, diminished self-worth, and identity confusion. The study also highlights that a lack of clear communication from coaches exacerbates these negative emotions and can strain both team dynamics and players’ personal lives. Furthermore, the results indicate that prolonged periods of exclusion may result in decreased training effort and a decline in overall performance, creating a negative feedback loop. These findings underscore the importance of transparent communication and supportive coach-athlete relationships in mitigating the adverse effects of reduced playing time. The study contributes to the literature by providing new insights into the underexplored area of athletes’ psychological responses to exclusion and offers practical recommendations for coaches to foster athlete motivation and well-being, even in challenging circumstances.

## Introduction

Given football’s team-oriented nature, players aspire to maximise their time on the field. This aspiration is fuelled by the potential rewards, including financial incentives and fame, that come with increased playing time, intensifying their competitive drive. Players aspire to maximise their time on the field [1–3]. However, when players’ expectations of playing time are unmet, they often experience dissatisfaction and may manifest these emotions in their interactions within the sports context. This is particularly pertinent to exceptional football talents on the global stage. Despite the universal desire among players to remain on the field for the entire 90-minute match duration, the actual allocation of playing time is at the discretion of the technical team and the coach.

### Communication, conflict, and the Coach-athlete relationship

The preferences of the technical team (the group of coaches and staff responsible for training, tactics, and team selection) and the head coach may cause the player to feel conflicted. Injury, burnout, lack of communication, and uncertainty are among the variables that cause conflict in sports [4]. From a practical perspective, understanding conflict management could provide a resource that allows athletes (and coaches) to enhance the quality of their sporting relationships [5]. Furthermore, the coaching style and coaching behaviours can help to overcome the adverse effects of specific game circumstances, such as being a substitute or losing a game, on athletes’ perceived justice of the coach [6,7]. Although it is the decision of the technical team who will be on the field, who will leave the game at which minute, who will sit on the bench, and who will enter the game later, the fact that the athlete is adequately informed about these issues affects the quality of the relationship between the coach and the athlete.

In football teams, for the coaches to keep the whole team’s performance at the highest level, they should be able to explain the reasons for their preferences sufficiently and ensure that the players trust them. Driscoll [8] explores the coaching style preferences of soccer athletes, emphasising the importance of coaches being knowledgeable, approachable, and open to input from players. The findings of Cranmer and Buckner [9] suggest that to promote functional patterns of athlete dissent, coaches must build quality relationships with athletes and create climates that facilitate cohesion among athletes. Wachsmuth, Jowett, and Harwood [10] explored conflict management approaches used by high-performance coaches and athletes to minimise dysfunctional and maximise functional outcomes of interpersonal conflict, revealing that coaches and athletes prevent the onset of conflict by facilitating good-quality relationships and optimal working environments, engaging in active conflict prevention strategies, and managing conflict by using intra- and interpersonal strategies, as well as by seeking out external help. When football players lose trust in coaches’ preferences, the coach-athlete relationship quality will decrease, and conflict will arise [11–13]. Athletes’ attributions about coach preferences gain importance to keep the quality of the coach-athlete relationship at the optimal level and to reduce conflict. The relationship is more robust when athletes perceive their coaches as competent and believe in their decisions [14,15].

### Attribution theory in sport psychology

Since soccer players may perceive this as a failure (low performance, failure to meet expectations, etc.) when they are not included in the squad and games or leave it unexpectedly, they may make negative attributions to these preferences. It is essential to understand the experiences of athletes who are substituted, who do not play, and who are out of the squad in football teams to develop a quality coach-athlete relationship and to realise initiatives that will contribute to the coaches taking measures to keep athlete motivation at an optimal level.

Scientific research shows that the quality of the coach-athlete relationship reduces conflict, appropriate conflict management strategies create a healthy sports environment for athletes [10,13,16], and accordingly, athletes trust coaches’ decisions and choices. This may affect athletes’ attributions about their performance and the coach’s behaviour. However, no studies in the literature investigate the experiences of footballers who appear less in the competition after playing for a long time. Understanding these players’ causal attributions about themselves points to an important issue that can profoundly impact athletes’ motivation, performance, and coach-athlete relationships. This gap indicates the need for a new perspective in sport psychology and training practice. In this context, this research was conducted to understand the causal attributions of soccer players towards themselves and how these attributions affect their sports experiences. This study aims to understand how soccer players evaluate these experiences and the effects of these experiences on themselves when they do not play. The research also seeks to expand the insights on this topic through the views of soccer players and the analysis of these views. This research can help soccer players better understand their periods of not playing and improve coach-athlete relationships. It can also provide better information on how coaches can improve athletes’ motivation and performance. Therefore, this study is expected to contribute to both the academic literature and the practical field of sport.

### Present study

More research needs to be done in the existing body of research about the experiences of athletes whose playing time diminishes based on coach preferences. While studies exploring the coach-athlete relationship have garnered significant attention and have substantially enriched our understanding of the sporting environment [17–20], there needs to be more investigation into how the reduction in playing time affects this relationship. Moreover, little attention has been given to understanding the variables that athletes attribute to the decline in playing time. The coach-athlete relationship has been extensively studied and acknowledged as a critical factor in sports, influencing performance, motivation, and overall well-being. However, the specific dynamics that emerge when athletes experience a decrease in their playing time remain relatively uncharted territory within the academic landscape. It is imperative to delve deeper into this unexplored domain to comprehensively grasp the ramifications of such experiences on athletes’ psychological states, behaviours, and overall performance milieu.

This study’s original contribution lies in its focus on unravelling the intricacies of the coach-athlete relationship when playing time is curtailed and identifying the variables that athletes attribute to a specific condition. By addressing this significant gap, this research aims to shed light on an underrepresented facet of sports psychology and offer valuable insights into the coach-athlete dynamic under circumstances of reduced playing time. This exploration promises to advance our understanding of athlete experiences, coaching practices, and the broader context of sports performance.

### Empirical evidence on attribution theory

In sports psychology, attribution theory is a primary topic in explaining causal attributions and their dynamics in sports [21,22]. Many social psychologists have proposed and modified various theories to make sense of the causal attributions of athletes. In addition, some researchers have reconceptualised attribution theories [23,24]. However, Weiner’s [25,26] theory is the approach that will enable us to understand best the players’ perceptions about the reasons for not playing and how they interpret and explain the coach’s decisions.

Studies on the attribution theory have revealed that while emphasising that the most fundamental reason for their relative success is themselves, individuals believe that their failures are due to reasons originating from the outside world. Moreover, it has become a scientific fact that individuals tend to exaggerate their positive aspects [27]. Since attribution theory deals with the causes of success and failure in achievement-oriented environments [28] and focuses on interpersonal behaviours and attitudes from the perspective of social psychology, sport provides a suitable environment for qualitative and quantitative analyses of causal attributions.

Sports-related results provide a logical test environment for understanding the psychological basis of the “ why “ question [29]. Attribution theory focuses on the reasons put forward to explain results, such as success and failure [28]. While one of the main components of Weiner’s [25,26] theory is emotions, another is the reasons attributed to success and failure [30]. Exercise and sports psychology researchers have published many analysis results that try to explain the relationship between coach and athlete in the sports environment based on attribution theory. Allen [21] published a systematic review of attribution studies conducted in the sports environment between 1954 and 2011. Studies involving motor tasks that were not performed in a performance setting and had little relevance to the sporting environment were excluded. 99.4% of the 167 studies conducted until 2011 were designed quantitatively. In Allen’s study, personal and trait attributions, natural environments, outcome bias, youth sports, testing of Weiner’s model, and intervention-type studies were included. These studies mainly focused on self-serving attribution biases, attribution and self-efficacy, attribution and emotions, attribution retraining, scale development, attribution, and performance. Some studies on attributions for success and failure have examined these attributions in terms of experiences of winning and losing [31–33]. Experienced and talented athletes and those with high self-efficacy may be more prone to self-serving bias in their attributions [34]. For example, Bond, Biddle, and Ntoumanis [35] determined a positive relationship between locus of causality and stability and self-efficacy. Athletes whose activity increased from pre-competition to post-competition made more stable and internal attributions for their performance than athletes whose activity decreased. Saarinen et al. [36] found that over the three-year period, a responsible attributional style—where individuals accept responsibility for both their successes and failures—predicted higher subsequent grade point averages and lower rates of sport dropout among student-athletes, even after accounting for their previous academic performance, gender, and type of sport.

### Application of attribution theory in football

While players acknowledge the coach’s pivotal role in this decision-making process, they can become increasingly critical of these decisions as their time on the field diminishes. This is especially true for football players who are relegated to substitute roles or excluded from the team entirely. In this context, attribution theory emerges as a valuable framework for comprehending the experiences of football players who find themselves excluded from the squad due to coaching decisions. It enables us to delve into the cognitive processes through which players ascribe reasons and meanings to their playing time or lack thereof, shedding light on the intricacies of the coach-athlete relationship in such scenarios. Therefore, the purpose of this study is to investigate the attributions made by football players regarding their reduced playing time, their perception of the underlying causes of this decision, and how these perceptions impact their overall experiences in sports. Furthermore, the study aims to understand better how coaches can enhance communication and support to maintain the quality of coach-athlete relationships, even when difficult decisions about playing time are made.

## Materials and methods

### Research model

This study employed thematic analysis, a recognised qualitative research methodology [37,38]. Thematic analysis provides a flexible and systematic approach to analysing qualitative data, enabling researchers to identify, organise, and interpret patterns or themes within the data [39,40]. This research’s primary objective is to investigate participants’ perceptions and experiences through their lenses. This approach allows for a detailed exploration of the unique and multifaceted aspects of human experience, focusing on how participants articulate their emotions, describe their experiences, and construct meaning from them. Thematic analysis is particularly well-suited for studies aiming to understand subjective realities and uncover the underlying themes that shape participants’ perspectives [41,42].

### Ethical statement

Based on the principles of research ethics, this study received the necessary institutional ethics approval from the University’s Ethical Committee, denoted as decision number 10/210017, issued on the 3rd of November 2021. Before their inclusion in the research, players were formally approached and presented with a detailed explanation of the study’s objectives and procedures. Subsequently, their informed consent was obtained through a verbal agreement. Notably, all athletes who met the predetermined inclusion criteria exhibited a willingness to participate in the research, thereby providing their voluntary and informed consent to partake in interviews as facilitated and conducted by the authors.

### Participants

The criterion sampling technique, a recognised purposeful sampling method, was employed to select research participants. This technique involves the deliberate selection of participants based on specific criteria predetermined by the researcher. In this study, the participants (the ages ranged between 23–27) were football players chosen according to a particular set of criteria for their performance in matches conducted between the 2021–2024 football season in Turkey’s top football league.

The study’s inclusion criteria encompassed football players featured in fewer than half of the 18 matches in the starting eleven or were introduced as substitutes later in the game, subsequently not being part of the initial lineup or substitution roster. Within these particular seasons, 16 football players met these predetermined inclusion criteria.

Additionally, all participants held the distinction of having competed for the Turkish National Team at some point across various levels of international play, reflecting a high level of proficiency and experience in football.

### Data collection, coding, and creating themes

A semi-structured interview approach was judiciously selected [43]. The choice of the semi-structured interview method was underpinned by its capacity to allow participants to articulate and elucidate their subjective experiences, thereby enabling them to convey the intricacies of their cognitive perceptions.

The interview sessions were conducted in a singular session, with an average duration of approximately 10–15 minutes each. Participants were presented with three meticulously formulated questions during these interviews to elicit rich and reflective responses. This approach encourages participants to offer in-depth insights into their experiences, facilitating a nuanced exploration of the themes under scrutiny.

The formulated interview questions, which served as the instruments for data collection and participant engagement, are as follows:

1. What are the reasons for your being excluded or benched?
2. What/how did you feel when you were excluded or benched?
3. How did this affect you?

### Analysis

Thematic analysis was preferred for analysing the data obtained in the study. A deductive approach was employed in the thematic analysis. The thematic analysis consists of six stages [41,42]:

1. Becoming familiar with the data and identifying potential relevant items
2. Generating initial codes
3. Searching for themes
4. Reviewing potential themes
5. Defining and naming themes
6. Producing the report

Following this sequence, the data were first read carefully. Then, the researcher sought answers to the research questions within the data. At this stage, the researcher became familiar with the data while simultaneously setting boundaries around the research questions. The key concepts related to the research questions were the reasons for not playing, the feelings associated with not playing, and the effects of not playing. The potential items to be coded within the data were read and identified within the context of these key concepts. In the ongoing process, the researcher listed the key concepts in the data. Listing these key concepts served as a roadmap for the researcher in the coding and codebook development stages. Based on these key concepts, a codebook was prepared, outlining the definitions of the codes and when they should be used. Identifying key concepts does not imply that the researcher engaged in deductive coding. This is because the emergence of codes occurs during the coding process. Here, the researcher only delineates an area where coding will be performed based on the research questions. Subsequently, notable expressions in the data were coded, and related codes were grouped and categorised. From this point onward, the researcher began searching for themes and subthemes. While reviewing and refining the themes, the coded data segments were revisited and verified that the themes were meaningful and consistent. Tables 1–3 illustrate identifying codes from participants’ statements, categorising the relevant codes, and developing themes.

**Table 1:** Thematic analysis process of the “attributions to being excluded” theme.

Statements/Quotes	Code	Sub-Theme/Category	Theme
*“...The coach told me they wanted to make a change in the team’s game plan, but I couldn’t understand this decision...” (Player 8)*	Changes in game plan	Tactical Reasons	Attributions to Being Excluded
*“Initially, I was playing regularly, and my performance was good. However, at some point, the coach started benching me consistently. This happened suddenly, and no clear explanation was given. The coach only told me it was a ‘tactical’ decision.” (Player 13)*	Tactical changes		
*“At first, I was in good form and even played a key role in some matches. However, as the season progressed, I was suddenly benched. The coach said they wanted to try a different system and implied that there was no place for me in this system.” (Player 14)*	Experimenting with a different system		
*“...In one match, I was suddenly benched and subsequently kept on the sidelines. I don’t believe there was a decline in my performance, but the coach simply didn’t prefer me...” (Player 4)*	Not being preferred by the coach	Coach’s Personal Preferences	
*“Over time, I realised it was more than just a tactical decision. The coach told me to change my playing style, but I think I was benched for personal reasons. There was no decline in my performance; other factors were at play.” (Player 6)*	Personal reasons		

**Table 2:** Thematic analysis process of the “emotions that arise” theme.

Statements/Quotes	Code	Sub-Theme/Category	Theme
*“I felt excluded. It was as if I wasn’t part of the team. You work hard every week to make the lineup, but it’s disappointing when you don’t see your name. At first, I thought it was a temporary decision, but as weeks passed, I realised it could be permanent, and it wore me down mentally.”* (Player 2)	Feeling excluded, feeling unvalued	Exclusion and Lack of Worth	Emotions that arise
*“...With each week on the bench, my morale dropped. I felt unvalued and began questioning my place in the team, which hurt my self-esteem. Sitting on the sidelines after working so hard was incredibly challenging.”* (Player 1)			
*“At first, I found it hard to make sense of the situation. I wasn’t injured, I was performing well in training, but I still couldn’t play. It was deeply disappointing for me.”* (Player 4)	Sense of uncertainty, lack of understanding	Uncertainty and Incomprehension	
*“Like every player, I initially thought being benched was a temporary situation. But as it became permanent over the weeks, my morale was severely impacted. What bothered me most was the lack of a clear explanation for why I wasn’t playing. I was constantly told I needed to be ‘better,’ but never what exactly needed improvement. This heightened my sense of uncertainty. I also started feeling excluded in some way. Being left out repeatedly undermined my confidence, weakened my belief in myself, and diminished my motivation.”* (Player 6)			
*“When you’re not on the field, you feel like something is missing. As a footballer, you express yourself on the pitch, and when you’re not playing, it feels like you’ve lost part of your identity.”* (Player 3)	Identity loss, loss of motivation	Loss of Motivation and Confidence	
*“When you’re consistently on the bench, you eventually lose your motivation. I started feeling drained both physically and mentally. Even though I continued to give my best in training, each week I wasn’t in the squad chipped away at my confidence.”* (Player 8)			

**Table 3:** Thematic analysis process of the “effects of being benched or excluded” theme.

Statements/Quotes	Code	Sub-theme/Category	Theme
*“My self-confidence was seriously damaged. Every day you go to training, but on match day, you don’t even see your name on the squad list. This is very demoralising. Since I believed my performance was still good, I kept asking myself why I wasn’t playing. This process was mentally very challenging, but I didn’t give up. Thanks to the support of my family and friends, I tried to keep my spirits high. Not playing lowered my motivation, but it also made me more determined.”* (Player 1)	Low self-confidence, Demoralisation, Self-questioning, Low motivation	Mental Effects	Effects of Not Playing
*“My motivation was seriously affected. I put in more effort every day to play, but being on the bench every week took a toll on my morale. When you’re not on the pitch, you don’t feel like part of the team. This shook my self-confidence.”* (Player 4)			
*“... During this period, I decided to seek professional support because I felt I needed help to overcome this situation psychologically. My life outside of football also started to be affected. My interest in training decreased, and my performance in training dropped, which negatively affected me even more.”* (Player 5)	Decreased interest in training, Decreased training performance	Effects on Physical Performance	
*“I struggled mentally throughout this process. I felt I wasn’t being given the chance to show myself on the pitch. This lowered my motivation, and I couldn’t perform well enough in training. I carried the stress of this situation at home too. I even had frequent arguments with my family.”* (Player 17)	Stress reflected at home, Arguments with family	Effects on Life Outside the Field	
*“The longer I stayed out, the less interested I became in training. I used to give my full energy to every session, but now I attended reluctantly. I started to distance myself from my teammates. I even began to feel detached in the locker room. This also affected my daily life. I started to experience more tension with my family, and I lost the joy I used to get from football.”* (Player 13)	Distance from teammates, Negative impact on daily life, Tension in life		

## Findings

In this dedicated section, the study comprehensively presents the thematic analysis results, elucidating the salient themes and codes that emerged from the data. The analysis offers a profound exploration of the nuanced experiences of football players who were observed to be inactive participants during the study. These experiences are encapsulated within three overarching thematic categories, each offering distinctive insights into the multifaceted nature of non-participation in football.

The first thematic category delves into the “Attributions to being excluded,” shedding light on the multifarious factors and circumstances contributing to a player’s non-participation in matches. This theme underscores the contextual and situational elements that influence a player’s presence on the field, encompassing team strategy, player performance, or unforeseen contingencies.

The second theme, “Emotions that arise,” delves into the emotional landscape of football players who find themselves on the sidelines. This theme offers a profound examination of the complex emotional states experienced by players when they are not actively engaged in matches. It seeks to understand the spectrum of emotions, ranging from frustration and disappointment to motivation and determination, that individuals may encounter during these periods of non-participation.

The third thematic category explores the “Effects of being benched or excluded.” It delves into the implications and ramifications of being relegated to the bench or not participating in matches. This theme unveils the broader consequences for a player’s career, team dynamics, and personal development, providing a comprehensive understanding of the impact of non-participation in football.

These themes, meticulously extracted from the data and supported by rigorous analysis, provide a comprehensive framework for understanding the intricate experiences of football players when they are not actively participating in matches. Thus, they contribute to an enriched comprehension of the dynamics and complexities inherent in professional football.

The attributions of the football players who participated in the study were categorised into two sub-themes (Table 1): “Tactical Reasons” and “Coach’s Personal Preferences.” Under the sub-theme “Tactical Reasons,” the players’ statements during the interviews were coded with tactical changes, tactical necessity, tactical preferences, changes in the game system, experimenting with a different system, and tactical decisions. Players generally reported starting the season well but later finding themselves excluded from the starting lineup without fully understanding why, attributing this to tactical reasons. Player 10’s statements support this view:

*“I started the season well, even scoring in a few matches. However, suddenly, the coach began keeping me on the bench. After a few matches, I was completely excluded from the squad. I don’t believe there was a significant drop in my performance, so I found it difficult to understand this decision. Tactical explanations were provided, but I didn’t think it was a performance-related issue. That’s why I started considering other reasons for being benched”* (Player 10).

As Player 10 emphasised, the players insisted that their exclusion was unrelated to their performance. On the contrary, they stated that they were not given playing time despite no decline in their performance.

*“At the start of the season, I was in form and played the full 90 minutes in many matches. But after some time, I found myself on the bench. The coach made tactical changes and tried a different strategy, but in my opinion, the issue wasn’t tactical. My performance was fine, but I was suddenly overlooked. The coach didn’t communicate with me, so I struggled to understand the situation”* (Player 16).

The second sub-theme identified as a reason for not playing is “Coach’s Personal Preferences.” The codes used for this sub-theme included not being preferred by the coach, personal reasons, not entirely tactical, individual or other factors, and seeking other explanations. Players expressed that their lack of playing time was not solely due to tactical reasons but also the coach’s personal decisions. Similar sentiments were echoed by Player 14:

*“Initially, I was in good form and even played a key role in some matches. However, as the season progressed, I was suddenly benched. The coach said he wanted to try a different system and implied that there was no place for me in this system. But in my view, my exclusion wasn’t just about tactical changes. I couldn’t understand why I was benched for such a long time. I wasn’t physically unfit or injured, but with every match, I felt more and more sidelined”* (Player 14).

All players participating in the study were asked why they were not playing and attributed their exclusion to external factors. They did not associate their absence with their own performance. Even Player 17, who had experienced an injury, stated that despite recovering and regaining his performance level, the coach still did not include him in the squad:

*“Before my injury, I was playing regularly, but after recovering, I couldn’t reclaim my place. The coach gave me a few opportunities as a substitute, but I never returned to the main squad. This was very disappointing for me. I had overcome my injury and was physically ready, but I still lost my place. I couldn’t make sense of why I wasn’t playing”* (Player 17).

The feelings of football players when they are not selected to play were examined under three sub-themes: *Exclusion and Worthlessness*, *Uncertainty and Incomprehension*, and *Loss of Motivation and Self-Confidence*.

In the sub-theme of *Exclusion and Worthlessness*, codes such as feeling worthless, feeling excluded, distancing oneself from the team, and not feeling like part of the team were identified from the players’ statements during the interviews. Players expressed that the prolonged time of not being selected made them feel disconnected from the team and excluded. Although they initially perceived this situation as temporary, the thought that it might become permanent led them to experience more negative emotions.

*“I felt excluded. It was as if I was no longer part of the team. You work hard every week to earn your spot, but when you don’t see your name on the team sheet, it’s disappointing. At first, I thought it was just a temporary decision, but as the weeks went by, I realised it might be permanent, and that mentally drained me”* (Player 2).

Some players even reported feeling worthless as the situation dragged on. Supporting this view, Player 5 shared:

*“Not playing had a significant mental impact on me. As a footballer, you want to be on the pitch. Sitting on the bench is demoralising. Week after week of not being selected made me feel worthless”* (Player 5).

The second sub-theme, *Uncertainty and Incomprehension*, encompassed feelings of ambiguity and a lack of clear explanation. Codes such as lack of clarity, inability to understand, and feelings of uncertainty were prominent. Some players stated that they were not informed why they were not being selected, leaving them unable to make sense of the situation. Player 6 expressed this sentiment as follows:

*“At first, like every player, I thought being on the bench was a temporary situation. But as the weeks went on, and it became a long-term issue, my morale was seriously affected. What troubled me most was the lack of a clear explanation as to why I wasn’t being played. I was constantly told, ‘You need to be better,’ but it was never made clear what exactly needed improvement. This increased my sense of uncertainty. I felt excluded in a way, and being left out repeatedly also shook my self-confidence. I lost faith in myself, and my motivation dwindled”* (Player 6).

The third sub-theme, *Loss of Motivation and Self-Confidence*, revealed that players experienced a decline in their drive and belief in themselves as the situation persisted. Codes such as low motivation, loss of identity, diminished confidence, feelings of inadequacy, and reduced self-assurance were identified. While players initially tried to stay motivated, the prolonged duration of not playing led to a significant loss of motivation, contributing to lowered self-confidence. Supporting this, players shared the following views:

*“At first, I thought it was temporary. I tried to motivate myself, thinking it was just a one-match decision and that I’d be back soon. But as the weeks went on, things changed. Each week I was left on the bench, my morale dropped. I started to feel worthless and questioned my place in the team, which shook my self-esteem. After putting in so much effort, sitting on the sidelines was very hard”* (Player 1).

*“When you’re constantly on the bench, you eventually lose your motivation. I started to feel physically and mentally drained. I was still giving my all during training, but with every week that I wasn’t included in the squad, my self-confidence diminished. I stopped feeling like part of the team”* (Player 8).

*“Every week I was left out, I felt lonelier. I missed being part of the team, feeling the pre-match excitement, and fighting on the pitch. The longer I stayed out, the more I felt an emptiness inside that I couldn’t suppress. Feeling like I’d been pushed to the sidelines is every professional footballer’s worst nightmare. My self-confidence was shattered, and I constantly felt like I had done something wrong”* (Player 14).

The responses from the football players to the question of how not playing affected them have been examined under three sub-themes: “mental effects,” “effects on physical performance,” and “effects on life outside the field.” In the sub-theme of mental effects, expressions used by the players during the interviews were coded using terms such as low self-confidence, low motivation, and self-questioning. It was observed that players who were unable to play were particularly affected negatively in terms of mental health. Even though they tried to do their best in training, they felt demoralised when they saw that they were not included in the squad or not starting. This led them to question the decision and themselves, ultimately negatively impacting their self-confidence. The following quotes support this view:

*“It was a very tough process mentally. I was constantly questioning myself. I put in more effort in training, hoping to catch the coach’s attention. But as I stayed on the bench week after week, I began to feel that my efforts were in vain. I can say that this harmed my self-confidence. In the end, not being on the pitch is not only physically but also psychologically challenging”* (Player 2).

Another factor affecting the players when they did not play, the second sub-theme, was the effects on physical performance. The codes used for this sub-theme included decreased interest in training, reduced training performance, and decreased physical performance. It was observed that mental effects were reflected in physical performance. Factors such as reduced motivation and decreased self-confidence led to players becoming less willing to participate in training, which subsequently had a negative impact on their training performance and, therefore, their physical performance. The following quotes support this:

*“Not playing was a very challenging process, not just physically but also mentally. You don’t get the opportunity to prove yourself, and this seriously affects your morale. My self-confidence was shaken, and my interest in training decreased. While I used to enjoy being on the pitch, now every day felt like a struggle...”* (Player 7).

The third sub-theme, which examines the effects of not playing, is “effects on life outside the field.” Codes used for this sub-theme included arguments with family members, tense living, negative impact on daily life, and feelings of distance from teammates. It was observed that the effects of not playing did not remain confined to the players’ inner worlds but also negatively affected their family lives and normal life outside of football.

*“As I stayed on the bench, my self-confidence decreased. At first, I thought being benched was only temporary, but as this situation became permanent, my interest in training decreased. I struggled mentally, and when I couldn’t get the chance to show myself on the pitch, my desire to improve my performance also diminished. This reflected negatively on my life outside of football. I became stressed, and this harmed my personal relationships”* (Player 12).

Particularly, issues with family and close circles were mentioned:

*“This situation also affected my home life. I had tension in my relationships with my family because the joy I once got from football had completely disappeared”* (Player 16).

Upon closer inspection, it is evident that all three sub-themes are interconnected. Mental effects trigger both performance and the effects on life outside the field. Some players mentioned experiencing significant psychological difficulties in this process and sought professional support. One such player stated:

*“As I stayed on the bench, I started working even harder to improve because, at some point, I thought I would get back on the pitch. However, as I remained on the bench every week, my expectations began to fade, replaced by frustration. The decreased attention from the coach on me began to cause anxiety about my future. Mentally, I struggled a lot. I decided to seek professional support in this process because I realised, I needed help to overcome this situation psychologically. My life outside of football also began to be affected. My interest in training dropped, my performance in training decreased, and this further negatively impacted me”* (Player 6).

## Discussion

The findings from this study reveal insightful perspectives on the reasons football players attribute their exclusion from matches, the emotional impact it has on them, and the subsequent effects on their mental and physical well-being and their personal lives. The analysis uncovered two key reasons players attributed to their exclusion from the starting lineup: “Tactical Reasons” and “Coach’s Personal Preferences.” In both categories, players expressed that their exclusion was largely driven by external factors, rather than a perceived decline in their own performance.

The theme “Attributions to Being Excluded” revealed that players often struggled to comprehend their exclusion, attributing it to tactical reasons rather than performance-based issues. These players consistently reported starting the season in good form but suddenly found themselves benched without clear communication from the coach. One player noted that despite believing their performance was adequate, tactical explanations were given without a clear link to their actual performance, leaving them confused and questioning other potential causes for their exclusion. This finding aligns with previous research that suggests players may often feel sidelined due to changes in tactical strategies or the coach’s personal preferences rather than objective performance metrics.

However, there was also a notable subgroup of players who believed their exclusion stemmed from the coach’s personal preferences, further deepening the situation’s emotional impact. This aligns with the “coach-athlete relationship quality”, where the dynamics between coach and player can lead to exclusion based on subjective preferences rather than purely tactical or performance considerations. These attributions highlight a disconnect between players’ perceived effort and actual involvement, further exacerbating uncertainty and frustration.

The emotional consequences of exclusion were profound, as revealed under the theme “Emotions that arise.” Players reported a range of negative emotions, including feelings of exclusion, low self-worth, confusion, and a significant loss of motivation and confidence. As one player expressed, the uncertainty of not knowing whether the exclusion was temporary or permanent created a mental burden that gradually drained their morale. This sense of exclusion led to a loss of identity for some players, as they struggled to reconcile their self-image as athletes with their sidelined role within the team.

The findings suggest that exclusion from the squad, especially without clear communication, triggers a process of identity loss, where players begin to question their place within the team and the broader footballing community. These emotional responses are consistent with the literature, which identifies feelings of inadequacy and lowered self-esteem as common outcomes for athletes who face exclusion from play. The loss of motivation and identity, particularly when prolonged, can undermine not only players’ emotional well-being but also their future engagement in the sport.

The consequences of exclusion extended beyond emotional distress and had tangible effects on both players’ physical performance and their daily lives. The theme “Effects of being benched or excluded” highlighted the interconnectedness between mental and physical well-being. The mental effects, such as low self-confidence and decreased motivation, significantly impacted players’ physical performance, leading to reduced effort in training. This downward spiral is concerning, as it may hinder players’ ability to regain their position or perform at the required level when called upon.

Moreover, the exclusion process appeared to spill over into players’ personal lives, as tensions with family members and a general sense of disconnection from teammates were frequently reported. The mental strain from not playing seemed to affect players’ off-field relationships, highlighting the far-reaching consequences of exclusion in sports. In some cases, players sought external psychological support, demonstrating the significant toll that this experience can have on their mental health.

In success or failure cases, causal attributions reveal an essential link between motivation and emotions [25]. Studies show that athletes tend to use internal attributions when they are successful and external attributions when unsuccessful [44]. In the study, it was determined that the athletes considered not playing as a failure. Coach preferences were seen as the reason for this failure. Attribution theory suggests that outcome and attributional evaluations affect emotion formation. The outcome evaluation automatically assesses performance regarding perceived success and failure. Based on this performance appraisal, general positive or negative emotions, including happiness and sadness, arise depending on the outcome. Outcome evaluation is followed by identifying perceived reasons for success or failure, called attributions, which are considered primary determinants of discrete emotional experience [45]. Britton [46] found a relationship between the athlete’s attribution style and the coach’s behaviours. It was found that there is a negative relationship between internality and democratic behaviour and a positive relationship between stability and positive feedback. Athletes and coaches exhibit distinct responses to success and failure. Success typically breeds heightened self-assurance, while failure often triggers pessimistic ruminations [47]. Football serves as a vivid arena illustrating these emotional reactions. For instance, when a footballer wins a match, there’s usually an increase in self-confidence and joy derived from the team’s success. However, losing a match can instigate a cascade of negative thoughts and emotions within a footballer’s mind.

Coping mechanisms for failure encompass problem-focused, emotion-focused, appraisal-focused, and avoidance-focused responses, with athletes experiencing significant motivational shifts following failure [48]. Football, as a sport, exemplifies these coping strategies. After a defeat, players might engage in introspection, seeking solutions to rectify their performance, or they might channel their emotions through training harder or altering their game tactics.

Moreover, the concept of perfectionism magnifies the adverse emotions associated with failure. High levels of self-oriented and socially prescribed perfectionism predict increased feelings of shame and guilt in athletes [49]. In football, this can manifest when a player excessively fixates on their mistakes, leading to a diminished sense of self-worth or confidence on the field.

Fear of failure also substantially impacts athletes, influencing their well-being, interpersonal interactions, and overall performance. This fear prompts athletes to adopt various coping strategies [50]. In football, fear of failure might cause players to play conservatively, avoid taking risks on the field, or exhibit hesitancy in critical game moments, impacting the team’s overall dynamics and success.

In football, the reactions to success and failure provide a compelling narrative about the intricacies of human psychology and resilience, showcasing how these emotional responses can significantly influence individual performance and team dynamics. In this research, the attributions of football players toward the coach trigger devaluation, decreased physical effort, stress, and excessive anxiety. The prolonged periods of not playing and the fact that the coaches’ preferences were the most obvious reasons for these revealed feelings of anger and sadness later in the process. Studies have shown that outcome evaluations interact with causal attributions in distinguishing emotions such as pride, gratitude, and anger [51]. In addition, the lack of apparent reasons for not playing, that is, the coach does not give a satisfactory explanation, reduces the athletes’ efforts. Therefore, football players who do not understand the reason for not being on the team experience confusion and psychological unrest [52]. Conflict may ensue depending on the coach’s preference when there is no apparent justification for the athletes not playing—that is, failure. The most obvious reason for this conflict is the feelings that cause the athlete to feel worthless, inadequate, and excluded. Rejeski, Darracott, and Hutslar [53] suggested that the difference between coach and athlete attributions can lead to conflicts that “can be catalytic to negative consequences of an evaluative, motivational, and behavioural nature.” In one of the recent studies on attribution in sport, Prosoli et al. [54] examined the Croatian combat athletes’ attribution patterns for their successes and failures. They found that athletes emphasised psychological factors as key to both success and failure, with a larger focus on psychological aspects in failure instances compared to success.

Footballers become uneasy when they think there is an inconsistency between the coach’s decision and their beliefs (ability, physical and mental effort) that they have the necessary conditions to play. Cooper [55] stated that individuals might become nervous when they cannot consistently change their beliefs about the consequences of events. Therefore, football players began to doubt their abilities and sufficient effort, experience career and future uncertainties, and finally think they were devalued, depending on the causal attributions associated with the coach. According to Försterling [56], indecision about causal inferences is related to feelings of self-doubt, uncertainty, and worthlessness.

When the attributions of the football players are examined, it is seen that they refer to various emotions, they think about leaving the team, and they tend to make compulsory decisions regarding their careers. The obligation to make decisions regarding their careers may be due to the emotions triggered by attributions. In addition, it is seen that football players tend to reduce their physical efforts significantly. Rejeski and Lowe [57] stated that athlete emotions could affect career decisions, training methods, and competition perspectives.

Considering causal inferences of the football players, it is seen that they are willing to make realistic attributions about their current situations and environments. Before deciding about their failure, football players use the information they have obtained from the coach’s choice. For example, football players tended to evaluate their physical condition, trainer preference, and the performance of their teammates before deciding that they were unsuccessful. Försterling [56] emphasised that individuals are motivated to evaluate information rationally and use the information presented to them for causal attribution before deciding about the reasons for performance outcomes.

According to the attribution theory, there are three causal dimensions [25,26]: Locus of causality, stability, and controllability. The locus of causality is associated with whether the cause originates from the individual or a situation outside the individual [29]. The locus of causality in the players’ attributions was the coach. Therefore, most of the attributions were based on the coach’s decisions. Stability is related to the expectation that the cause will change or remain fixed over time [25,58]. Players who did not play for a long time and considered their condition a failure lost their belief that this situation would change over time and started to think that it had become a stabilised condition caused by the coach. They began to believe that they had to leave their environment. When evaluated in terms of controllability, related to the fact that the cause is due to a source other than the person making the attribution or the individual [45], the footballers initially believed they could control the situation with physical effort.

According to the players interviewed, their coaches do not have enough information about the current situation of these athletes. It is evident that the coach-athlete relationship has a vital role in the attributions of these athletes. Studies show that athletes and coaches have different attributions regarding situational factors [59]. This may be due to the possibility that athletes better evaluate their situation. The coach can make inferences about the athlete’s physical condition, but it is more challenging to predict mental conditions. The coach-athlete relationship can be a key factor in mediating the misunderstandings between athletes and coaches about the decisions that football players consider failures. Athletes may accept their coach’s decision when the quality of the relationship between coach and athlete is high.

## Conclusion

Since a significant part of football players’ performance depends on the coach’s approach to the athlete, the relationship between the coach and the athlete is essential in keeping the performance of a football player who does not play at an optimal level. Because players’ motivation and performance will be affected by the coach’s attitude towards the athlete, interpersonal relationship processes and conflict resolution strategies [60] used within the attribution theory framework can offer serious solutions to the problems between the coach and these athletes. At this point, it should be noted that coaches can keep all their players mentally and physically ready by clearly explaining their preferences to their athletes. If the football players evaluating their performance do not have enough information and constantly make false inferences, that is, attributions, they will have a high tendency to experience learned helplessness. According to Prapavessis and Carron [61], tennis players experiencing learned helplessness gave internal, permanent, and repetitive scores in the attribution dimensions. Thus, it supports the suggestion that those with learned helplessness have low evaluations of their ability to control and change the factors contributing to poor performance. Maier and Seligman [62] stated that learned helplessness tends to increase when false attributions persist. Preventing false attributions may be possible by preventing athletes from misinterpreting the information. An explanation of the decision made by the coach can enable the athletes to understand the information and shape their attributions correctly. Rees et al. [24,63] stated that athletes might not perceive all the information presented to them by their coaches and teammates, ignore some of the feedback or distort the importance of the feedback.

Clear explanations to the athletes about their coach choices can prevent coach-athlete conflict. Encouraging communication between the coach and the athlete will not only enable the athlete to understand the coach’s preferences but also help the coach to understand the reasons for the athlete’s behaviour. Consistency, distinctiveness, and consensus information suggested by Rees et al. [24] can be used to overcome the negative impact of misattributions, which turn into behaviours or are common in the players participating in our research. Future research should examine the role of social interaction in formulating the attributions of football players. Social expectations are effective in the athlete’s attributions to coach preferences and failure. Since social effects will create attribution bias [64], the evaluations of the attributions shaped by family, manager, teammate, and fan expectations of football players are important in terms of team sports, where keeping the athlete’s performance at an optimal level contributes to team performance. Such research can provide important information to coaches and athletes, especially sports psychologists, in enhancing team performance.
